# Correction: The impact of financial development on enterprise green innovation under low carbon pilot city

**DOI:** 10.1371/journal.pone.0308345

**Published:** 2024-07-31

**Authors:** Jianxiao Du, Yajie Han, Xiaoyu Cui

The funding statement for this paper is incorrect. The correct funding statement is as follows: This research was generously supported by the China Postdoctoral Science Foundation (Grant No. 2023M734089), the Guangdong Province Philosophy and Social Science Foundation Youth Project (Grant No. GD24YYJ09), and the Zhuhai Philosophy and Social Science Foundation (Grant No. 2023YBB032).

The affiliations for the first and second authors are also incorrect. Jianxiao Du and Yajie Han are not affiliated with both #1 and #2. Instead, Jianxiao Du is affiliated with #1 and Yajie Han is affiliated with #2. The correct affiliations are as follows:

Jianxiao Du^1^, Yajie Han^2^, Xiaoyu Cui^3^

**1** School of Accountancy, Shandong University of Finance and Economics, Jinan, Shandong Province, China, **2** Zhuhai Fudan Innovation Institute, Hengqin, China, **3** School of Economics, Southwestern University of Finance and Economics, Chengdu, China.

The first two authors, Jianxiao Du and Yajie Han, should be noted as contributing equally to this work.
