# Supplementary material for: The impact of financial development on enterprise green innovation under low carbon pilot city
Source: PLoS One. 2024 Jun 25;19(6):e0301909. doi: 10.1371/journal.pone.0301909 (PMC11198762; doi:10.1371/journal.pone.0301909)
Supplement: Attachment — Submitted filename: Review Comments.pdf [file pone.0301909.s001.pdf]

The manuscript entitled "The Impact of Financial Development on Enterprise Green Innovation under Low Carbon Pilot City" provides an empirical analysis on studying the influence of financial development on the green innovation of enterprises in low-carbon pilot cities. The authors mainly used double difference and multiple fixed effects models to examine the real effect of financial development on the green innovation of enterprises. Also, the authors further explore underlying mechanisms through which financial development can affect green innovations of enterprises, and found that it contributes to relax financing constraints and trigger R&D investment. Overall, the manuscript is well-written and interesting. I have several minor comments and suggestions for the authors:

1. It is believed that the ***Low Carbon Pilot City*** policy is not a randomized controlled experiment in the strict sense. Thus, I would recommend the authors to elaborate the reason why the study chooses the low-carbon pilot cities as the data sample instead of other similar environmental regulation pilots polices, such as ***Smart City Pilot, Green Finance Reform and Innovation Pilot***, ***National-Innovation City Pilot***.

2. The current abstract has too lengthy words, and the description to the research significance appears too redundant. Thus, I would recommend the authors to re-organize the research background, research methods, research findings and research significance using a more concise way.

3. The research gap is not identified in a compelling way in the introduction section. *IMPORTANCE, NOVELTY* should be clearly stated and presented in the introduction section.

4. The study documented supportive evidence that the crucial role of financial development in attaining sustainable development goals, and this finding is highly consistent with the connotation of green finance of environmental finance. Thus, I would recommend the authors to add one significant contribution of this study in green

finance or environmental finance by citing the following recent impactful research to connect to this ongoing discourse.

[1] https://doi.org/10.1016/j.techfore.2022.122007

[2] https://doi.org/10.1016/j.jbusres.2023.114147

[3] https://doi.org/10.1016/j.strueco.2022.10.007

5. Comparisons with other studies have to be provided in the DISCUSSION section. Please interpret and describe the significance of your findings in light of what was already known about the research problem being investigated and explain any new understanding or fresh insights about the problem after you've taken the findings into consideration. Please provide a comparison with other studies.

6. Provide detailed background and a rationale for methodology in subsection 3.3. The METHODOLOGY SECTION of your paper should clearly articulate the reasons why you chose A PARTICULAR PROCEDURE OR TECHNIQUE. The methodology should discuss the problems that were anticipated and the steps you took to prevent them from occurring, such as the standard commend trend and placebo tests in running the DID regression.

Good luck!
