# Supplementary material for: The impact of financial development on enterprise green innovation under low carbon pilot city
Source: PLoS One. 2024 Jun 25;19(6):e0301909. doi: 10.1371/journal.pone.0301909 (PMC11198762; doi:10.1371/journal.pone.0301909)
Supplement: Attachment — Submitted filename: Response to Reviewers.docx [file pone.0301909.s002.docx]

*Dear Reviewer,*

*On behalf of my co-authors, we greatly appreciate your insightful questions and comments that have helped us to significantly improve the quality of our manuscript entitled “PONE-D-23-22886”. According to your comments, we carefully revised our manuscript. The comments from you and our responses are as follows.*

**Responds to the reviewer’s comments:**

| **Reviewer：** | |
| --- | --- |
| 1. Thank you for updating your data availability statement. You note that your data are available within the Supporting Information files, but no such files have been included with your submission. At this time we ask that you please upload your minimal data set as a Supporting Information file, or to a public repository such as Figshare or Dryad.  Please also ensure that when you upload your file you include separate captions for your supplementary files at the end of your manuscript.  As soon as you confirm the location of the data underlying your findings, we will be able to proceed with the review of your submission. | **Response:**  Thank you very much for your feedback and guidance. We have now uploaded the data as a "Supporting Information" file, and added the relevant supplementary file captions at the end of the manuscript, as per your recommendations. These modifications have been completed, and we hope this meets your requirements.  We appreciate your attention to our research and the valuable insights you've provided. As soon as the data upload and revisions are finalized, we will promptly confirm the location of the data underlying our findings to proceed with the review process smoothly. |
| 2. Please ensure that you refer to Figure 3 & 4 in your text as, if accepted, production will need this reference to link the reader to the figure. | **Response:**  Thank you for your valuable feedback. We have made the necessary updates to the manuscript by referring to Figure 3 and Figure 4 in the corresponding text. These references have been included to facilitate readers' access to the figures.  We sincerely appreciate your guidance, and we hope that these modifications align with your expectations. Should you have any further comments or suggestions, please do not hesitate to let us know. Your input is greatly appreciated. |
| 3.Please ensure that you refer to Table 1, 12 and 14 in your text as, if accepted, production will need this reference to link the reader to the Table. | **Response:**  Thank you for your feedback. We have carefully revised the manuscript to include references to Table 1, 12, and 14 in the appropriate sections of the text. These references are now in place to ensure that readers can easily access the corresponding tables.  Your input has been invaluable in improving the quality of our manuscript, and we appreciate your thorough review. |

*Once again, thanks for your constructive suggestions! We tried our best to improve the manuscript and made some changes in the manuscript. These changes will not influence the content and framework of the paper. And here we did not list the changes but used the red notation format to make the changes in revised paper.*

*We look forward to hearing from you!*

*Kind regards,*

*Jianxiao Du*

*Xioayu Cui*
