# Supplementary material for: The impact of financial development on enterprise green innovation under low carbon pilot city
Source: PLoS One. 2024 Jun 25;19(6):e0301909. doi: 10.1371/journal.pone.0301909 (PMC11198762; doi:10.1371/journal.pone.0301909)
Supplement: Attachment — Submitted filename: Response to Reviewers.docx [file pone.0301909.s004.docx]

*Dear Reviewer,*

*On behalf of my co-authors, we greatly appreciate your insightful questions and comments that have helped us to significantly improve the quality of our manuscript entitled “PONE-D-23-22886”. According to your comments, we carefully revised our manuscript. The comments from you and our responses are as follows.*

**Responds to the reviewer’s comments:**

| **Reviewer：** | |
| --- | --- |
| 1. Generally, the abstract of a paper is based on research aim/purpose, research method,  and key findings. Abstract of this paper is well written but it is required to highlight the  key findings of the study. Please revamp the abstract and make sure to include  information as below, in order:  - Motivation  - Objective  - Data and method  - Results  - Implications | **Response:**  Thank you very much for your review and valuable feedback. Based on your suggestions, I have made revisions to the abstract to better highlight the key findings of the study. I hope these modifications meet your expectations. If there are any other areas that require improvement, please feel free to let me know. I appreciate your patient guidance. |
| 2. The Introduction fails to motivate the study. In the present form, it resembles a minireview of literature, rather than discussing any policy-level problem. Why this study is necessary? What policy level problem this study is addressing? How is the study expected to provide any solution to that problem? How does the choice of sample is complementing that problem? Are the results and policies generalizable? The introduction is silent in all these aspects. The mere choice of new variables, new methods, or choosing a new context is not considered as contribution of a study. In the introduction section, the study should be positioned within the context of more contemporary literature. In this direction, more recent literature can be used to motivate the research question adequately. Meanwhile, the authors are strongly advised to derive the gap in which the study intends to fill from the existing literature. This section thus requires a thorough revision. Please check this paper: Estimating the trade-environmental quality relationship in SADC with a dynamic heterogeneous panel model. African Review of Economics and Finance 13(1): 113-165 | **Response:**  Thank you very much for your thorough review and valuable feedback. In accordance with your suggestions, we have undertaken a comprehensive revision of the introduction section, taking inspiration from the provided article "Estimating the trade-environmental quality relationship in SADC with a dynamic heterogeneous panel model" to better motivate the study and align it with contemporary literature.  We deeply appreciate your guidance, which is crucial for enhancing the quality of our research. Please review the modifications we have made, and if there are any further areas that need improvement, we would be more than willing to make additional adjustments. Thank you for your patience and professional advice. |
| 3.Originality: Structurally, this paper is well-written with well-established econometric methods. However, the most critical issue that is grossly lacking in this paper is the motivation of this paper. Hence, the background of the study should be strengthened with the issue centred on global per-capita CO2 emissions, and it should be well justified why it is important to carry out this study. | **Response:**  Thank you for your thorough review and valuable feedback. In response to your suggestion, we have strengthened the background of the study, focusing on the issue of global per-capita CO2 emissions. We have provided detailed justification for the importance of conducting this study. |
| 4.The contributions of the paper are very weak. What are the contributions of the document to the empirical literature? The authors can show how this study differs from other studies and also elaborate more on the contributions of this paper. Addition of more recent literature will make the work more relevant to readers. | **Response:**  Thank you for your review and valuable feedback on our paper. In response to your suggestion, we have provided a detailed explanation of how this study differs from other relevant research and further emphasized the contributions of our work. Additionally, we have incorporated more recent literature to ensure that our study remains current and relevant to readers' expectations. |
| 5. The paper should be restructured. Section 2 should attempt to summarize the empirical literature. This section needs to be rewritten and strengthened. The authors are advised to divide this section into three sub-sections. The first part should clearly illustrate the theoretical studies linking the variables under review. The second section should concentrate on empirical works between these variables. The last section should summarize the literature gaps. What is the aim of the review of literature? The authors should not merely list out the studies without even creating a debate among them. Without that debate and thoughtful contradictions, the research gap cannot be substantiated. Also, the current literature appearing in this section should be strengthened. The authors should use more recent studies in this section. Meanwhile, more can be done to reflect more comparison in the literature against other regions. For instance, in Europe, BRICS, Africa and Asia. To improve this section, the authors are invited to use the following papers and cite them.   a) https://doi.org/10.3389/fenvs.2022.1044605  b) https://doi.org/10.1007/s43621-022-00117-3  c) https://doi.org/10.1186/s40854-023-00453-x  d)https://doi.org/10.1007/s41247-023-00110-y  e) https://doi.org/10.1186/s40854-022-00396-9  f) https://doi.org/10.1007/s11356-022-21107-y  g)https://doi.org/10.1007/s11356-019-05944-y  h)https://doi.org/10.1080/12265934.2019.1695652  i) https://doi.org/10.1007/s10644-020-09285-6  j) https://doi.org/10.1007/s11356-021-17193-z  k)https://doi.org/10.1007/s10644-021-09368-y  l) https://doi.org/10.3390/su141610268  m)https://doi.org/10.1080/13504509.2022.2123411  n) https://doi.org/10.3389/fenvs.2022.985719  o) https://doi.org/10.1002/sd.2473  p)https://doi.org/10.1080/13504509.2023.2183526  q) https://doi.org/10.21203/rs.3.rs-419113/v1  r) https://doi.org/10.1002/sd.2535  s)https://doi.org/10.1080/27658511.2023.2210950  t) https://doi.org/10.1002/sd.2597  u) https://doi.org/10.1002/sd.2618  v)https://doi.org/10.1080/19463138.2023.2222264  w) https://doi.org/10.1002/sd.2473  x) https://doi.org/10.3280/EFE2022-002006  y)https://doi.org/10.1080/23311886.2023.2234230  z) https://doi.org/10.1016/j.wds.2023.100096  aa)https://doi.org/10.1007/s41247-023-00112-w  bb)https://african-review.com/online-first-details.php?id=69 | **Response:**  Thank you for your thorough review and valuable suggestions. In response to your feedback, we have restructured the literature review section into three subsections to clearly present theoretical studies, empirical works, and identified gaps in the literature.  We have included the references you provided and integrated additional recent literature to enhance the timeliness of the literature review. We believe these modifications better meet your expectations. If there are further areas for improvement or additional suggestions, we would be more than willing to make adjustments. Thank you once again for your professional guidance and patient review. |
| 6.The authors neglect the significance of the study in the introduction section. Why? Several studies have been conducted regarding this topic at hand; therefore, it is crucial for the investigators to incorporate the novelty as well as the significance of the study. | **Response:**  Thank you very much for your review and valuable suggestions regarding our study. We genuinely appreciate your guidance and take your advice on the introduction section seriously. We have already emphasized the novelty and significance of our research in the introduction, making efforts to convey the uniqueness of our study to the readers. We will revisit the introduction section to ensure a clear communication of the research's importance, and we are committed to making necessary modifications to better meet your expectations. |
| 7.In section 3 (Data Description and Model Design), after reorganizing the paper, the authors should provide a clear theoretical underpinning before the empirical framework. Please, restructure this section with a clear theoretical framework demonstrating how the variables under review are related. The theoretical underpinning between these variables should be properly justified in this sub-section. The authors should provide a justification of the use of the variables chosen. Please adjust accordingly by incorporating that. The authors should use the above-mentioned papers to strengthen this section. | **Response:**  Thank you for reviewing our paper and providing valuable feedback. We highly appreciate your suggestion to provide a clear theoretical foundation in Section 3 (Data Description and Model Design). Following your guidance, we have elaborated on the explanatory and dependent variables, striving to present a more explicit theoretical framework that demonstrates the relationships between these variables. |
| 8.Authors are advised to conduct the robustness check to strengthen the paper. The authors should use some of those papers mentioned above to strengthen this sub-section. | **Response:**  Thank you for your thoughtful feedback and suggestion regarding the robustness checks in our paper. We would like to mention that in our study, we have conducted a robustness analysis of the regression results using three different approaches: substituting the dependent variable with a proxy variable, altering the policy implementation batches, and introducing policy lag terms. Additionally, we have performed parallel and placebo tests to ensure the robustness of our results. We believe these checks provide a comprehensive examination of the stability of our findings.  However, we acknowledge your recommendation to further strengthen this sub-section by incorporating relevant papers mentioned above. We will carefully review and integrate relevant literature to enhance the robustness checks in our paper. Your input is valuable, and we are committed to making the necessary revisions to address your suggestion. |
| 9.The authors merely reported the results without even discussing the economic intuitions behind the results. Are these results supporting or refuting the existing policies in the chosen context? Are the results directed towards any new policy initiatives? The discussion of results should open up the threads of policy discussion, which is completely absent in this case. A mere comparison of the results with the literature doesn't ensure the novelty of the results unless they give out something new on the theory/policy front. Moreover, the discussion of results should be properly tied to past literature, and emphasis should be placed on how past studies either support or refute the findings of this study and why. Therefore, this section needs revision. | **Response:**  Thank you very much for your valuable feedback on our paper. We fully understand your concerns, especially regarding the lack of economic intuition behind the results and the need for a more in-depth policy discussion. We will carefully consider the issues you raised, particularly in terms of the relationship between our results and existing policies, and whether they support any new policy initiatives. We are committed to making substantial revisions to the results discussion section to address these points. Your suggestion to closely tie our results to past literature is well taken. We will ensure that our paper seamlessly integrates with previous studies, emphasizing how they either support or refute our findings and providing clear explanations for any discrepancies. |
| 10.To improve the quality of this manuscript, the authors are invited to use the papers already mentioned above and some useful ones in the area to strengthen the introduction, literature review, methodology and results and discussion sections. | **Response:**  Thank you very much for the valuable suggestions regarding our research. We have carefully considered and implemented your advice by incorporating the previously mentioned references and other relevant studies in the field to strengthen the introduction, literature review, methodology, and results and discussion sections. Through this process, we are confident that the quality of the paper has been further enhanced, and it now better integrates previous scholarly contributions. |
| 11.The policy implications of the results need more substantiation than what is currently stated in the paper. | **Response:**  Thank you very much for your thorough review of our research and for providing valuable feedback. We have carefully considered your suggestions regarding the need to strengthen the policy implications of our study. To enhance the substantiation of policy insights, we have implemented the following improvements:  Detailed elaboration in the discussion section on specific policy recommendations, emphasizing the foundation and logic behind these suggestions.  Connection of our research findings with existing policy frameworks, highlighting their positive impact on practical policy-making and indicating potential areas for improvement. |
| 12.Conclusion reiterates the results, which is completely undesirable. The author(s) should summarize the results within a maximum of 3 sentences. The authors should kindly strengthen this section. Moreover, the policies are completely vague, and it seems that the authors already had the policies in mind before even starting the paper. The policies should be directly derived from the discussion of the results, and they should not go beyond the results. The policy implications of the results need more substantiation than what is currently stated in the paper. The policy suggestion section needs improvement. Kindly improve it. | **Response:**  Thank you for revisiting our paper and providing valuable feedback on the conclusion section. We have made the requested modifications to ensure that the conclusion now succinctly summarizes the results within a maximum of three sentences. We appreciate your guidance on strengthening this section and hope that the changes meet your expectations.  Regarding the policy implications, we have addressed the concern about vagueness and ensured that the policies are directly derived from the discussion of results without going beyond them. Furthermore, we have substantiated the policy suggestions with more detailed reasoning to enhance the overall quality of the policy implications section.  We sincerely appreciate your patience and thoughtful guidance throughout the review process. If you have any additional suggestions or if further improvements are needed, we are more than willing to make the necessary adjustments. |
| 13.Kindly improve the study limitation(s) and possible direction for future research after the policy recommendation section. | **Response:**  Thank you for your constructive feedback on the study limitations and possible directions for future research after the policy recommendation section. We have carefully revised this portion of the paper in accordance with your suggestions and hope that the changes address your concerns.  We have taken the opportunity to enhance the clarity and depth of our discussion on study limitations, providing a more comprehensive acknowledgment of the constraints inherent in our research. Additionally, we have expanded on potential directions for future research, offering more nuanced insights and avenues for further investigation.  We appreciate your guidance in refining these sections and value your input in improving the overall quality of our manuscript. If you have any further recommendations or if there are additional aspects that require attention, we are eager to make any necessary adjustments. |
| 14.Finally, it is vital that this manuscript is proofread by a native speaker of English language to further strengthen easy readership. | **Response:**  Thank you for your thorough review and valuable feedback on our manuscript. We appreciate the time and effort you have dedicated to providing constructive comments.  We have taken your suggestion seriously and conducted a comprehensive proofreading of the manuscript to enhance its readability. However, we acknowledge that there may still be some areas where language organization could be improved. We apologize for any remaining shortcomings and are committed to making further refinements to ensure the manuscript meets the highest standards of clarity.  Your input has been invaluable in the improvement process, and we sincerely thank you for your guidance. If you have specific areas or suggestions for language enhancement, we would greatly appreciate your insights to make the necessary adjustments. |

*Once again, thanks for your constructive suggestions! We tried our best to improve the manuscript and made some changes in the manuscript. These changes will not influence the content and framework of the paper. And here we did not list the changes but used the red notation format to make the changes in revised paper.*

*We look forward to hearing from you!*

*Kind regards,*

*Jianxiao Du*

*Xioayu Cui*
