# Supplementary material for: The impact of financial development on enterprise green innovation under low carbon pilot city
Source: PLoS One. 2024 Jun 25;19(6):e0301909. doi: 10.1371/journal.pone.0301909 (PMC11198762; doi:10.1371/journal.pone.0301909)
Supplement: Attachment — Submitted filename: Response to Reviewers.docx [file pone.0301909.s005.docx]

*Dear Reviewer,*

*On behalf of my co-authors, we greatly appreciate your insightful questions and comments that have helped us to significantly improve the quality of our manuscript entitled “PONE-D-23-22886”. According to your comments, we carefully revised our manuscript. The comments from you and our responses are as follows.*

**Responds to the reviewer’s comments:**

| **Reviewer：** | |
| --- | --- |
| 1.Thank you for submitting your manuscript to PLOS ONE. In a previous round of review, Reviewer 3 included several requests to cite specific works - these requests were in points 5a-bb of the comments in the decision letter. We understand that you have added citations to several of these works in your revised manuscript. Please note that it is not necessary or expected to cite the works requested by the reviewer. We apologise that this was not noted in the previous decision letter.  In light of this, we are issuing this revision decision to provide you an opportunity to remove the citations added in response to the previous reviewer's request. You may remove all or none of the references, at your discretion. No further revisions are required before your manuscript proceeds to acceptance. | **Response:**  Thank you very much for your feedback and guidance. I have removed some of the references that were added in response to the requests from the reviewer, as you suggested. We sincerely appreciate your advice and will proceed with further modifications accordingly.  Please feel free to let us know if there are any other adjustments or revisions needed. We look forward to advancing this manuscript to the next stage.  Once again, thank you for your assistance and support. |

*Once again, thanks for your constructive suggestions! We tried our best to improve the manuscript and made some changes in the manuscript. These changes will not influence the content and framework of the paper. And here we did not list the changes but used the red notation format to make the changes in revised paper.*

*We look forward to hearing from you!*

*Kind regards,*

*Xioayu Cui*

*Jianxiao Du*
