# Supplementary material for: The impact of financial development on enterprise green innovation under low carbon pilot city
Source: PLoS One. 2024 Jun 25;19(6):e0301909. doi: 10.1371/journal.pone.0301909 (PMC11198762; doi:10.1371/journal.pone.0301909)
Supplement: Attachment — Submitted filename: Referee Report.pdf [file pone.0301909.s003.pdf]

RECOMMENDATION: MAJOR REVISION

Manuscript Number: PONE-D-23-22886R1

The Impact of Financial Development on Enterprise Green Innovation under Low Carbon Pilot City

The paper attempts to investigate how financial development influences enterprise green innovation in low carbon pilot city (LCPCs). To achieve this, the authors used data from listed companies in various cities in China between 2010 and 2018 and found that LCPC policies stimulate institutional green innovation within enterprises. Overall, the paper is interesting and exhibits good potential. However, it suffers from several deficiencies that need to be addressed before it can be accepted for publication.

1) Generally, the abstract of a paper is based on research aim/purpose, research method, and key findings. Abstract of this paper is well written but it is required to highlight the key findings of the study. Please revamp the abstract and make sure to include information as below, in order:
   - Motivation
   - Objective
   - Data and method
   - Results
   - Implications
2) The Introduction fails to motivate the study. In the present form, it resembles a mini-review of literature, rather than discussing any policy-level problem. Why this study is necessary? What policy level problem this study is addressing? How is the study expected to provide any solution to that problem? How does the choice of sample is complementing that problem? Are the results and policies generalizable? The introduction is silent in all these aspects. The mere choice of new variables, new methods, or choosing a new context is not considered as contribution of a study. In the introduction section, the study should be positioned within the context of more contemporary literature. In this direction, more recent literature can be used to motivate the research question adequately. Meanwhile, the authors are strongly advised to derive the gap in which the study intends to fill from the existing literature. This section thus requires a thorough revision. Please check this paper: Estimating the trade-environmental quality relationship in SADC with a dynamic heterogeneous panel model. *African Review of Economics and Finance 13*(1): 113-165

3) Originality: Structurally, this paper is well-written with well-established econometric methods. However, the most critical issue that is grossly lacking in this paper is the motivation of this paper. Hence, the background of the study should be strengthened with the issue centred on global per-capita CO_2 emissions, and it should be well justified why it is important to carry out this study.
4) The contributions of the paper are very weak. What are the contributions of the document to the empirical literature? The authors can show how this study differs from other studies and also elaborate more on the contributions of this paper. Addition of more recent literature will make the work more relevant to readers.
5) The paper should be restructured. Section 2 should attempt to summarize the empirical literature. This section needs to be rewritten and strengthened. The authors are advised to divide this section into three sub-sections. The first part should clearly illustrate the theoretical studies linking the variables under review. The second section should concentrate on empirical works between these variables. The last section should summarize the literature gaps. What is the aim of the review of literature? The authors should not merely list out the studies without even creating a debate among them. Without that debate and thoughtful contradictions, the research gap cannot be substantiated. Also, the current literature appearing in this section should be strengthened. The authors should use more recent studies in this section. Meanwhile, more can be done to reflect more comparison in the literature against other regions. For instance, in Europe, BRICS, Africa and Asia. To improve this section, the authors are invited to use the following papers and cite them.
    a) https://doi.org/10.3389/fenvs.2022.1044605
    b) https://doi.org/10.1007/s43621-022-00117-3
    c) https://doi.org/10.1186/s40854-023-00453-x
    d) https://doi.org/10.1007/s41247-023-00110-y
    e) https://doi.org/10.1186/s40854-022-00396-9
    f) https://doi.org/10.1007/s11356-022-21107-y
    g) https://doi.org/10.1007/s11356-019-05944-y
    h) https://doi.org/10.1080/12265934.2019.1695652
    i) https://doi.org/10.1007/s10644-020-09285-6
    j) https://doi.org/10.1007/s11356-021-17193-z
    k) https://doi.org/10.1007/s10644-021-09368-y
    l) https://doi.org/10.3390/su141610268
    m) https://doi.org/10.1080/13504509.2022.2123411

n) https://doi.org/10.3389/fenvs.2022.985719
o) https://doi.org/10.1002/sd.2473
p) https://doi.org/10.1080/13504509.2023.2183526
q) https://doi.org/10.21203/rs.3.rs-419113/v1
r) https://doi.org/10.1002/sd.2535
s) https://doi.org/10.1080/27658511.2023.2210950
t) https://doi.org/10.1002/sd.2597
u) https://doi.org/10.1002/sd.2618
v) https://doi.org/10.1080/19463138.2023.2222264
w) https://doi.org/10.1002/sd.2473
x) https://doi.org/10.3280/EFE2022-002006
y) https://doi.org/10.1080/23311886.2023.2234230
z) https://doi.org/10.1016/j.wds.2023.100096
aa) https://doi.org/10.1007/s41247-023-00112-w
bb) https://african-review.com/online-first-details.php?id=69

6) The authors neglect the significance of the study in the introduction section. Why? Several studies have been conducted regarding this topic at hand; therefore, it is crucial for the investigators to incorporate the novelty as well as the significance of the study.
7) In section 3 (Data Description and Model Design), after reorganizing the paper, the authors should provide a clear theoretical underpinning before the empirical framework. Please, restructure this section with a clear theoretical framework demonstrating how the variables under review are related. The theoretical underpinning between these variables should be properly justified in this sub-section. The authors should provide a justification of the use of the variables chosen. Please adjust accordingly by incorporating that. The authors should use the above-mentioned papers to strengthen this section.
8) Authors are advised to conduct the robustness check to strengthen the paper. The authors should use some of those papers mentioned above to strengthen this sub-section.
9) The authors merely reported the results without even discussing the economic intuitions behind the results. Are these results supporting or refuting the existing policies in the chosen context? Are the results directed towards any new policy initiatives? The discussion of results should open up the threads of policy discussion, which is completely absent in this case. A mere comparison of the results with the literature doesn't ensure the novelty of the results unless they give out something new on the theory/policy front. Moreover, the discussion of results should be properly tied to past literature, and emphasis should be

placed on how past studies either support or refute the findings of this study and why. Therefore, this section needs revision.

10) To improve the quality of this manuscript, the authors are invited to use the papers already mentioned above and some useful ones in the area to strengthen the introduction, literature review, methodology and results and discussion sections.
11) The policy implications of the results need more substantiation than what is currently stated in the paper.
12) Conclusion reiterates the results, which is completely undesirable. The author(s) should summarize the results within a maximum of 3 sentences. The authors should kindly strengthen this section. Moreover, the policies are completely vague, and it seems that the authors already had the policies in mind before even starting the paper. The policies should be directly derived from the discussion of the results, and they should not go beyond the results. The policy implications of the results need more substantiation than what is currently stated in the paper. The policy suggestion section needs improvement. Kindly improve it.
13) Kindly improve the study limitation(s) and possible direction for future research after the policy recommendation section.
14) Finally, it is vital that this manuscript is proofread by a native speaker of English language to further strengthen easy readership.
